# Retraction: The effects of 21 and 23 milimeter aortic valve prosthesis on hemodynamic performance and functional capacity in young adults

**Published:** 2014

This retracts the article “The effects of 21 and 23 milimeter aortic valve prosthesis on hemodynamic performance and functional capacity in young adults” in Vol. 30 No. 2 from page 356-360, which was published in final edited form because of ethical misconduct as one of the authors copied some portion from an article published in Turkish language which was detected later. The authors have been notified and agree with the retraction. Retracted on April 15, 2014.

Yener AU, Ozcan S, Budak AB, Genc SB, Ozkan T, Cicek OF. The effects of 21 and 23 milimeter aortic valve prosthesis on hemodynamic performance and functional capacity in young adults. Pak J Med Sci. 2014;30(2):356–360.

doi: http://dx.doi.org/10.12669/pjms.302.4468

